# Emerging Role of Functional Magnetic Resonance Spectroscopy (MRS) to Monitor Response to Kinase Inhibitors in Cancer

**DOI:** 10.33552/acrci.2024.04.000587

**Published:** 2024-07-05

**Authors:** Kavindra Nath, Pradeep K. Gupta, Mariusz A Wasik

**Affiliations:** 1 Department of Radiology, University of Pennsylvania, Philadelphia, PA, USA; 2 Department of Pathology, Fox Chase Cancer Center, Philadelphia, PA, USA

**Keywords:** Magnetic Resonance Spectroscopy, Mantle Cell Lymphoma, Bruton’s Tyrosine Kinase, Metabolites

## Abstract

Inhibitors of kinases involved in signaling and other intracellular pathways, have revolutionized cancer treatment by providing highly targeted and effective therapies. However, timely monitoring treatment response remains a considerable challenge since conventional methods such as assessing changes in tumor volume do not adequately capture early responses or resistance development, due to the predominantly cytostatic rather than cytotoxic effect of kinase inhibitors. Magnetic resonance spectroscopy (MRS) is a non-invasive imaging technique that can provide insights into cellular metabolism by detecting changes in metabolite concentrations. By measuring metabolite levels, MRS offers a means to assess treatment response in real-time, providing earlier indications of efficacy or resistance compared to conventional imaging modalities. Bruton’s Tyrosine Kinase (BTK) is a critical enzyme involved in B-cell receptor signaling. BTK inhibitors have been approved for the treatment of Mantle Cell Lymphoma (MCL) and other B-cell malignancies. Recent studies involving genome-scale gene expression, metabolomic, and fluxomic analyses have demonstrated that ibrutinib, an index BTK inhibitor, profoundly affects the key metabolic pathways in MCL cells., including glycolysis, glutaminolysis, pentose shunt, TCA cycle and phospholipid metabolism. Importantly, the effects of ibrutinib on MCL cells directly and proportionately correlates with their sensitivity to the drug. Consequently, changes in specific metabolite concentrations detectable non-invasively by MRS such as lactate and alanine reflecting mostly the status of cellular glycolysis and glutaminolysis, respectively, have emerged as potential biomarkers for predicting response and resistance of MCL cells to BTK inhibition, both in vitro and in vivo. Preparations to validate the utility of these biomarkers in clinical setting are under way. These studies may pave the way to monitor therapeutic response to kinase inhibitors also in other types of cancer.

## Introduction

Small-molecule kinase inhibitors have demonstrated remarkable clinical efficacy across various types of lymphomas and other malignancies [1]. These inhibitors target in a highly specific manner kinases that play critical roles in signaling pathways involved in cell proliferation, survival, and differentiation [1]. By inhibiting these kinases, the inhibitors disrupt aberrant signaling pathways that drive tumor growth, leading to therapeutic benefits. The dependence on standard tumor volumetric imaging for staging and assessing therapeutic response in lymphomas, including Mantle Cell Lymphoma (MCL), poses challenges when considering treatments with kinase inhibitors [2–5]. Kinase inhibitors often exert mainly cytostatic effects and, hence, they inhibit chiefly cell proliferation with induction of cell death being much less pronounced [3,5]. This results in greatly delayed changes in tumor volume detectable by conventional imaging techniques and, hence, late clinical assessment regarding effectiveness of the therapy. Of note, in addition to affecting cell signaling, kinase inhibitors impair also profoundly cell metabolism, as the early effect of their therapeutic activity [6–8]. While FDG PET/CT detects one aspect of cell metabolism, i.e. glucose uptake. However, this measure has not proven a reliable biomarker of tumor response to kinase inhibition [3,5], and this imaging method continues to be used almost exclusively for initial staging and monitoring response to treatment based on changes in the tumor volume [4,5]. Therefore, alternative imaging modalities and biomarkers are being explored to address these limitations and improve the assessment of response to kinase inhibitors in MCL and other lymphomas. Beyond FDG PET/CT, functional imaging techniques such as magnetic resonance spectroscopy (MRS) [6], diffusion-weighted imaging (DWI) [7], dynamic contrast-enhanced-magnetic resonance imaging (DCE-MRI) [8], and can provide insights into tumor biology, including changes in cellularity, perfusion, and metabolism induced by kinase inhibitors. MRS is emerging as particularly effective in detecting and monitoring biomarkers of response to kinase inhibition, as shown using MCL as cancer model and the Bruton’s tyrosine kinase (BTK) inhibitor ibrutinib, as index kinase inhibitor [6–8].

## BTK Inhibition in MCL and Other Lymphomas

The application of ibrutinib, the first-generation small-molecule BTK inhibitor and other, even more specific BTK inhibitors has significantly changed the treatment landscape for various B-cell lymphomas, including MCL [9–11]. BTK is a tyrosine kinase with a highly cell-specific expression pattern restricted to B-lymphocytes and a few other cell types, which makes it a very attractive therapeutic target. BTK plays a critical role in cell signaling by being activated by B-cell and, possibly, toll-like/IL-1 receptor complexes [10–15]. The ibrutinib has been approved for the treatment of MCL, Chronic Lymphocytic Leukemia (CLL), small lymphocytic leukemia (SLL), marginal zone lymphoma, graft vs. host disease, and it also shows activity against other types of B-cell lymphoma [16]. Other small-molecule, second and third generation BTK inhibitors including acalabrutinib, zanubrutinib, and pirtoibrutinib have also demonstrated exceptional efficacy. However, because roughly one third of MCL patients fail to respond to BTK inhibition upfront and essentially all responders eventually develop resistance to the drug, the methods to evaluate and monitor its effect on lymphoma cells are critical for optimizing personalized patient management [9–11,17]. Although some patients experience transient redistribution of lymphoma cells into peripheral blood, monitoring MCL response by cell count cannot be considered a reliable and universal biomarker. Furthermore, while detection of circulating tumor-derived mutated DNA in blood by “liquid biopsy” can be informative, it is also a late marker of response to kinase inhibitors [17], similar to volumetric imaging methods. Recent studies have used patient derived MCL cell lines with different sensitivities to evaluate the efficacy of ibrutinib in vitro using proton magnetic resonance spectroscopy (^1^H MRS) in order to address this crucial issue of reliable and prompt determination of kinase inhibitor effect on malignant cells and tumors [6]. By examining metabolic changes in response to ibrutinib treatment, particularly alterations in lactate, alanine, and choline concentrations, we can identify potential early and sensitive biomarkers of BTK inhibition in MCL and other lymphoma treatments [6].

Direct measurement of lactate by ^1^H MRS is impeded by high levels of lipid that often occur within or around tumor cells producing large resonances that obscure the lactate methyl peak. Our research group has pioneered a spectral editing technique that utilizes Hadamard slice selective multiple quantum coherence (HDMD-Sel-MQC) transfer enabling to overcome this problem of detecting lactate and alanine [18]. Accordingly, we have demonstrated the utility of this method in MCL xenograft models to detect and monitor early therapeutic response to BTK inhibition by measuring lactate and alanine metabolites concentrations [19].

## Metabolic Effects of BTK Inhibition I un MCL Cells

The studies using sensitive ^13^C MRS and LC-MS methods to detect fluxes in specific metabolic pathways, foremost glycolysis and glutaminolysis that are perturbed by ibrutinib [6]. These perturbations are associated with changes in expression of genes encoding metabolic enzymes involved in these pathways as well as their metabolic substrates and products (6,7). Such genome-wide gene-expression studies with human MCL cell lines differing in their sensitivity to BTK inhibition indicated that the inhibition impairs glycolysis, glutaminolysis, pentose shunt, TCA cycle, and phospholipid metabolism. Importantly, changes in metabolites related to some of these key pathways: lactate (typically reflects predominantly glycolysis), alanine (biomarker of glutaminolysis), and choline (indicator of lipid metabolism) can be detected by ^1^H MRS [6]. These observations created the opportunity to monitor response to BTK inhibition. Indeed, Lee et al. [6] have demonstrated that simultaneous decreases in lactate and alanine metabolites levels serve as a robust metabolic indicator of MCL cell response to the BTK inhibition using ibrutinib. This response was evident as early as 24 hours following exposure to the inhibitor of three ibrutinib-sensitive MCL cell lines. Interestingly, changes in cell number were only noticeable after 48 hours of ibrutinib exposure, suggesting that metabolic alterations precede observable effects on cell proliferation. Furthermore, the metabolic shifts observed after 24 hours of ibrutinib treatment correlated with changes in the cell cycle at the same time point. Noteworthy, in cell lines with poor responsiveness to ibrutinib, no significant metabolic changes were detected after 24 hours of exposure. Interestingly, after 48 hours of ibrutinib exposure marked changes in lactate levels were observed in these poorly responsive cell lines, while alanine levels remained unchanged compared to the control group [6]. These findings suggest that alanine holds promise as a potential marker for ibrutinib resistance, possibly reflecting increased dependence of these cells on glutaminolysis rather than glycolysis. Thus, a combined assessment of lactate and alanine levels could provide more accurate insights into the response to ibrutinib treatment than a measurement of either of these metabolites alone [6]. This premise gained further justification by Gupta et al. [20] using high-resolution ^1^H MRS-based imaging studies to show that BTK inhibitor ibrutinib indeed promptly affected in MCL-derived cell lines concentration of lactate and alanine but also choline and it did so strictly proportionately to the cell line sensitivity to the drug in regard to the degree of impairment of their growth . These findings indicated the potential of lactate, alanine, and choline to become accurate and early biomarkers of effective BTK inhibition in MCL cells.

## Monitoring of BTK inhibition in MCL in vivo

Using xenografts of the MCL-derived cell lines with various levels of sensitivity to ibrutinib, Nath et al. [19] assessed the feasibility to evaluate by imaging response to BTK inhibition in vivo. Inhibition of intra-tumoral concentrations of lactate and alanine was measured by ^1^H MRS with HDMD-Sel-MQC pulse sequence and choline by ^1^H MRS with STEAM (Stimulated Echo Acquisition Mode) pulse sequence. These experiments have showed that ibrutinib therapy results in an early and profound inhibition in concentrations of lactate, alanine and, less universally, choline in MCL tumors. Similar to the in vitro studies, degree of the metabolite concentration inhibition directly and fully correlated with the degree of suppression of the MCL tumor growth.

This finding strongly suggest that the ^1^H MRS-based imaging should permit detection and monitoring of BTK inhibition effect in MCL patients, with preparation for studies of this kind already under way.

## Conclusions

This review article provides a comprehensive insight into the rationale for and approaches to detect BTK inhibition in MCL cells, as the model for cancer therapy with kinase inhibitors. It explores the importance of understanding the cellular mechanisms of inhibitor activity, foremost the impact of cell signaling on cell metabolism. ^1^H MRS-based imaging emerges as a feasible and very attractive method to detect and monitor MCL response to BTK inhibition. It evaluates in a non-invasive, reliable and sensitive fashion changes in concentration of key metabolites: lactate, alanine and possibly choline. The same or similar MRS-based approaches may prove feasible and beneficial to detect and monitor therapeutic response to kinase inhibitors in various types of cancer.

## Abbreviations:

MCL: Mantle Cell Lymphoma
^1^H and ^13^C MRS: Proton and Carbon Magnetic Resonance Spectroscopy
BTK: Bruton’s Tyrosine Kinase

## References

[1] Roskoski R Jr, (2023) Properties of FDA-approved small molecule protein kinase inhibitors: A 2023 update. Pharmacol 187: 106552. 10.1016/j.phrs.2022.106552 36403719

[2] Cottereau AS, Meignan M, Nioche C, Capobianco N, Clerc J. (2021) Risk stratification in diffuse large B-cell lymphoma using lesion dissemination and metabolic tumor burden calculated from baseline PET/CT(dagger). Ann Oncol 32: 404–411. 33278600 10.1016/j.annonc.2020.11.019

[3] Ma WW, Jacene H, Song D, Vilardell F, Messersmith WA, (2009) [18F] fluorodeoxyglucose positron emission tomography correlates with Akt pathway activity but is not predictive of clinical outcome during mTOR inhibitor therapy. J Clin Oncol 27: 2697–2704. 19380450 10.1200/JCO.2008.18.8383 PMC2689846

[4] Milgrom SA, Rechner L, Berthelsen A (2021) The optimal use of PET/CT in the management of lymphoma patients. Br J Radiol 94: 20210470. 34415777 10.1259/bjr.20210470 PMC8553204

[5] Zanoni L, Bezzi D, Nanni C, Paccagnella A, Farina A, (2023) PET/CT in Non-Hodgkin Lymphoma: An Update. Semin Nucl Med 53, 320–351. 36522191 10.1053/j.semnuclmed.2022.11.001

[6] Lee SC, Shestov AA, Guo L, Zhang Q, Roman JC, (2019) Metabolic Detection of Bruton’s Tyrosine Kinase Inhibition in Mantle Cell Lymphoma Cells. Mol Cancer Res 17:1365–1377. 30862686 10.1158/1541-7786.MCR-18-0256 PMC8757533

[7] Revheim ME, Hole KH, Mo T, Bruland OS, Reitan E, (2022) Multimodal functional imaging for early response assessment in patients with gastrointestinal stromal tumor treated with tyrosine kinase inhibitors. Acta Radiol 63: 995–1004. 34171968 10.1177/02841851211027389

[8] Kim JH, Lee JW, Park K, Ahn MJ, Moon JW, (2021) Dynamic contrast-enhanced MRI for response evaluation of non-small cell lung cancer in therapy with epidermal growth factor receptor tyrosine kinase inhibitors: a pilot study. Ann Palliat Med 10: 1589–1598. 33302635 10.21037/apm-19-622

[9] Eyre TA, Cheah CY, Wang ML (2022) Therapeutic options for relapsed/refractory mantle cell lymphoma. Blood 139: 666–677. 34679161 10.1182/blood.2021013326 PMC9710495

[10] Jain P, Wang ML Mantle cell lymphoma in 2022 (2022) A comprehensive update on molecular pathogenesis, risk stratification, clinical approach, and current and novel treatments. Am J Hematol 97: 638–656. 35266562 10.1002/ajh.26523

[11] Shirley M (2022) Bruton Tyrosine Kinase Inhibitors in B-Cell Malignancies: Their Use and Differential Features. Target Oncol 17: 69–84. 34905129 10.1007/s11523-021-00857-8 PMC8783859

[12] Inamdar AA, Goy A, Ayoub NM, Attia C, Oton L, (2016) Mantle cell lymphoma in the era of precision medicine-diagnosis, biomarkers and therapeutic agents. Oncotarget 7: 48692–48731. 27119356 10.18632/oncotarget.8961 PMC5217048

[13] Ran F, Liu Y, Xu Z, Meng C, Yang D, (2022) Recent development of BTK-based dual inhibitors in the treatment of cancers. Eur J Med Chem 233: 114232. 35247756 10.1016/j.ejmech.2022.114232

[14] Szklener K, Michalski A, Zak K, Piwonski M, Mandziuk S (2022) Ibrutinib in the Treatment of Solid Tumors: Current State of Knowledge and Future Directions. Cells 11(8): 1338. 35456016 10.3390/cells11081338 PMC9032968

[15] Wang X, Kokabee L, Kokabee M, Conklin DS (2021) Bruton’s Tyrosine Kinase and Its Isoforms in Cancer. Front Cell Dev Biol 9: 668996. 34307353 10.3389/fcell.2021.668996 PMC8297165

[16] Alu A, Lei H, Han X, Wei Y, Wei X (2022) BTK inhibitors in the treatment of hematological malignancies and inflammatory diseases: mechanisms and clinical studies. J Hematol Oncol 15: 138. 36183125 10.1186/s13045-022-01353-w PMC9526392

[17] Wang ML, Rule S, Martin P, Goy A, Auer R, (2013) Targeting BTK with ibrutinib in relapsed or refractory mantle-cell lymphoma. N Engl J Med 369: 507–516. 23782157 10.1056/NEJMoa1306220 PMC4513941

[18] Pickup S, Lee SC, Mancuso A, Glickson JD (2008) Lactate imaging with Hadamard-encoded slice-selective multiple quantum coherence chemical-shift imaging. Magn Reson Med 60: 299–305. 18666110 10.1002/mrm.21659 PMC2701382

[19] Nath K, Gupta PK, Sen N, Orlovskiy S, Wang S, (2023) Metabolic detection of BTK inhibition in vivo using MCL xenograft models. In Proceedings of the 2023 Mantle Cell Lymphoma Workshop and/or the Spring SAB Meeting Chicago.

[20] Gupta PK, Nath K, Sen N, Orlovskiy S, Wang S, (2023) Detection of Metabolic Biomarkers of Response to Ibrutinib Therapy in Mantle Cell Lymphoma Cells. In Proceedings of the 2023 Mantle Cell Lymphoma Workshop and/or the Spring SAB Meeting, Chicago, USA.

